# Mastery is central: an examination of complex interrelationships between physical health, stress and adaptive cognition, and social connection with depression and anxiety symptoms

**DOI:** 10.3389/fpsyt.2024.1401142

**Published:** 2024-05-01

**Authors:** Huiyoung Shin, Chaerim Park

**Affiliations:** Department of Psychology, Jeonbuk National University, Jeonju, Republic of Korea

**Keywords:** depression and anxiety symptoms, stress, mastery, anhedonia, physical health, gender difference

## Abstract

**Background:**

Research has established the link between physical health, stress and cognition, and social connection with depression and anxiety. Nevertheless, an understanding of the comorbidity of depression and anxiety symptoms and their complex interrelationships with relevant factors remains still limited. This study investigated the complex pattern of interplay between depression and anxiety symptoms and pertinent physical, cognitive, and social factors and potential gender differences.

**Methods:**

Using a sample of 600 middle-aged men and women, depression and anxiety as well as physical health, perceived stress and mastery, and social connection were assessed. The network structure of depression and anxiety symptoms and risk and resilience factors were characterized by examining interrelationships and the centrality indices of Strength and Bridge Strength. Gender differences were examined using the Network Comparison Test.

**Results:**

Perceived stress and mastery were central bridge factors influencing comorbid depression and anxiety symptoms, and perceived stress, anhedonia, and mastery exhibited strong inter-connections to each other. The connections of physical health-anhedonia and sleep disturbance-irritability were stronger in men than in women, while social connection with family was linked to interpersonal problems only in women.

**Conclusions:**

The results underscore that prevention and interventions targeting reducing perceptions of stress and promoting mastery would prevent onset or recurrence of depression and anxiety symptoms among middle-aged men and women. Engaging in behavioral activities to maintain physical health and ensuring adequate sleep could be particularly beneficial for men in reducing overall symptom severity.

## 1 Introduction

Depression and anxiety are highly common disorders in the general population, are a major contributor to disability worldwide (1) and are often comorbid (2). Based on the nationwide statistics of South Korea, the prevalence of depression and anxiety for adults in 2021 was estimated to be 7.7% and 9.3%, respectively, and the number of individuals suffering from these internalizing disorders increased 35.1% between 2017 and 2021 (3). As with many psychiatric disorders, depression and anxiety, as well as their symptoms have high comorbidity, which leads to greater debility than for either alone (4). These debilitating disorders significantly impact daily functioning and quality of life (5). They are also associated with work impairment and loss of productivity and increase the risks of developing physical and psychiatric comorbidity as well as premature mortality (6, 7). Both depression and anxiety are more prevalent in women, with 2.1:1 and 1.6:1 ratio in women compared with men, and the prevalence rates are higher among adults in midlife compared to early adulthood, with the highest rates occurring in adults in their 60s (17%) and 50s (15.8%), respectively (8). Other medium- to high-income countries report similar results, suggesting there has been an increase in depression and anxiety symptoms between early adulthood and midlife, followed by a subsequent decline in old age (9–11).

Given the enormous burden and high occurrence of depression and anxiety during mid-adulthood, it is crucial to understand the factors that put middle-aged adults at risk for developing and maintaining these internalizing disorders. Much of the previous work has identified relevant individual and environmental factors that escalate or reduce the risks for development of depression and anxiety, that are suitable for screening (e.g., genetic liabilities; neuroticism; earlier life adversity) (2). Relatively fewer studies have investigated factors that can be changed by individuals without extensive intervention (12). Moreover, understanding of the co-occurrence of depression and anxiety symptoms and their interrelationships with individual and environmental factors remains still limited. High co-occurrence between depression and anxiety disorders suggests that there are shared underlying factors that may alleviate or increase comorbidity among symptoms of these disorders. In order to identify individuals who are susceptible to depression and anxiety disorders and to provide prevention efforts to reduce the incidence and burden of these disorders, it is crucial to understand central modifiable risk or resilience factors, and their interactions with depression and anxiety symptoms.

Conventional clinical practices and the Diagnostic and Statistical Manual of Mental Disorders 5th edition (DSM-5) (13) are based on the notion that symptoms of psychiatric disorders are indications of an underlying cause and various symptoms are equally important as diagnostic criteria to assess severity of depression and anxiety (14). Accordingly, the standard procedure to assess the severity of depression and anxiety is to sum up different rating scale symptom scores into one total score. However, depression and anxiety scales are multidimensional and using aggregated symptom scores could obscure important differences between heterogenous groups and weights of symptoms, as well as interrelationships among symptoms (14, 15). Concerns on the validity of using total scores to assess depression and anxiety have motivated the network approach (16), which presumes that psychiatric disorders are combinations and mutually reinforcing interactions of symptoms within a network. Based on this approach, a handful of studies have shown that the presence of certain symptoms can trigger other symptoms, and comorbidity of depression and anxiety disorders can result from active interactions between symptoms that are relevant to both disorders (15, 17). By examining symptom-level associations, multiple studies have also revealed the importance of some symptoms over others (i.e., central symptoms) and identified bridge symptoms that connect different disorders (15, 18, 19).

Network perspective is also a promising approach to examine the interrelationships between comorbid depression and anxiety symptoms and modifiable risk or resilience factors. As for psychiatric symptoms, various individual and environmental factors for depression and anxiety often coexist and interconnect with each other. Hence, it is necessary to consider the interdependent and interacting nature of different individual and environmental factors and depression and anxiety symptoms, as well as the different strength of associations among psychiatric symptoms and relevant factors (16). Research has underscored that moving beyond simply examining symptom-symptom interactions of psychiatric disorders and considering risk or resilience factors within network models is critical to shed light on the pathway to psychiatric disorders. Furthermore, this approach is required to elucidate the processes that protect against the development of psychopathology and to provide insights into the potential applications that provide targeted treatment (20, 21).

In this study, we included physical, cognitive, and social factors that have consistently been recognized as etiological or resilience factors for depression and anxiety disorders within the network model. Previous studies have provided strong evidence of longitudinal associations between physical illness and depression and anxiety symptoms as well as the high comorbidity between sleep disturbance, depression, and anxiety (22–24). Abundant research has also established the robust associations between perceived stress, (mal)adaptive cognitions, and depression and anxiety symptoms (25, 26). For instance, depression symptoms are precipitated by major stressful events and recurrent symptoms are triggered by subsequent minor stressors (27). Furthermore, (ma)adaptive cognitions have been considered important contributors to depression and anxiety symptoms in the face of stressful events (28, 29). Theoretical models and empirical evidence have suggested that depression and anxiety result from beliefs about worry and uncontrollability (30, 31), and such beliefs of control and sense of mastery are significantly linked with endorsement of depression and anxiety, as well as stress and sleep disturbances (29, 32). Moreover, extant literature corroborates the importance of the link between social connection with affective disorders (33, 34). Research has found that large social networks, frequent contact with social partners, and high levels of social participation have protective benefits and alleviate depression and anxiety. As such, there is a clear connection between physical health, perceived stress and cognition, and social connection with depression and anxiety symptoms. However, their interrelationships are likely to be highly complex, for which network analysis is suited to investigate the interacting nature of different factors and depression and anxiety symptoms.

Therefore, the goal of the present study was to examine the network structure of depression and anxiety symptoms and relevant physical, cognitive, and social factors to better understand how they interact with one another among middle-aged men and women. This group was chosen, as mid-adulthood, typically defined as 40–65 years (35), has been reported to be an at-risk time of life demonstrating high occurrences of depression and anxiety (8–11, 36). To elucidate which risk or resilience factors have most prominent roles in depression and anxiety symptoms, we detected central and bridge factors in the network. Further, given gender is known to be a significant influencing factor underlying prevalence rates of depression and anxiety (21, 37), we investigated potential gender differences in the overall network structure as well as the strength of associations among depression and anxiety symptoms and risk and resilience factors. Ultimately, by considering various individual and environmental factors in tandem with depression and anxiety symptoms, the present study provides important insights into the complex patterns of the interplay of depression and anxiety symptoms with risk and resilience factors, and provides evidence-based and gender-specific health-promoting strategies to reduce psychopathology symptoms and to better support psychological health.

## 2 Methods

### 2.1 Procedures

We recruited participants from an online research participant system that retains a panel of 1,724,264 South Korean adults across varying age, education, income, and demographic as well as clinical characteristics. We used stratified probability sampling to obtain a representative sample of middle-aged men and women. The invitation was distributed to those who qualified to fill one of 6 subgroups defined by three strata for age (40–49, 50–59, and 60–69 years) and two strata for gender (men and women) from February 15 to 17, 2023. To understand a fuller spectrum of psychological health among middle-aged adults, we adopted dimensional conceptualizations (38), that is, middle-aged adults with and without depression and anxiety symptoms were recruited so that the full spectrum of psychological health, from normal to abnormal, could be measured. Inclusion criteria were the following (1): South Korean adults living in South Korea (2); aged between 40-65 years (3); able to understand the aims and content of the survey. Ethical approval was obtained from the Institutional Review Board (IRB) of researchers’ University and informed consent was obtained from all of the participants.

### 2.2 Participants

The final sample comprised 600 middle-aged adults (mean age = 52.68; 48.17% male) and 299 (49.83%) of them reported either depression or anxiety symptoms. The prevalence of depression (CES-D ≥ 16) and anxiety (GAD-7 ≥ 5) were 44.00% and 35.83%, respectively. About 485 (80.84%) of the participants had received higher education (undergraduate/graduate school), 421 (70.17%) were currently employed, and 409 (68.16%) had income higher than $30,000. About 499 (83.17%) were married or partnered and 466 (77.67%) had at least one child. **Tables 1** and **2** provide more detailed demographic information and the information on the mean of CES-D and GAD-7, as well as physical, cognitive, and social factors that were included in the analysis.

**Table 1:** Demographic and clinical characteristics.

	Male (*n* = 289)		Female (*n* = 311)		Total (*N* = 600)		$\chi^{2}$	*p*-value
	*n*	%	*n*	%	*n*	%		
Age (years)							1.34	0.51
40–49	105	36.33%	104	33.44%	209	34.83%		
50–59	106	36.68%	110	35.37%	216	36.00%		
60–65	78	26.99%	97	31.19%	175	29.17%		
Education							14.09	<0.01
< High school	38	13.15%	77	24.75%	115	19.17%		
Undergraduate school	211	73.01%	204	65.59%	415	69.17%		
Graduate school	40	13.84%	30	9.65%	70	11.67%		
Marital status							11.91	<0.05
Unmarried	53	18.34%	42	13.50%	95	15.83%		
Married	219	75.78%	238	76.53%	457	76.17%		
Partnered	4	1.38%	1	0.32%	5	0.83%		
Divorced	11	3.81%	18	5.79%	29	4.83%		
Widowed	2	0.69%	12	3.86%	14	2.33%		
Income							17.75	<0.01
< $10,000	34	11.76%	64	20.58%	98	16.33%		
$10,000–$20,000	17	5.88%	26	8.36%	43	7.17%		
$20,000–$30,000	18	6.23%	32	10.29%	50	8.33%		
$30,000–$40,000	48	16.61%	50	16.08%	98	16.33%		
≥ $40,000	172	59.52%	139	44.69%	311	51.83%		
Depression (CES-D)							7.62	0.06
No depression (0–15)	157	54.33%	179	57.65%	336	56.00%		
Mild depression (16–20)	31	10.73%	41	13.18%	72	12.00%		
Moderate depression (21–24)	32	11.07%	16	5.14%	48	8.00%		
Severe depression (> 25)	69	23.88%	75	24.12%	144	24.00%		
Anxiety (GAD-7)							1.14	0.77
No anxiety (0–4)	185	64.01%	200	64.31%	385	64.17%		
Mild anxiety (5–9)	61	21.11%	68	21.86%	129	21.50%		
Moderate anxiety (10–14)	34	11.76%	30	9.65%	64	10.67%		
Severe anxiety (15–21)	9	3.11%	13	4.18%	22	3.67%		

**Table 2:** Descriptive information of depression and anxiety symptoms, as well as physical, cognitive, and social factors.

Variable	Total		CES-D				*t*	GAD-7				*t*
	(*N* = 600)		< 16 (*n* = 336)		≥ 16 (*n* = 264)			< 5 (*n* = 385)		≥ 5 (*n* = 215)		
	*M*	*SD*	*M*	*SD*	*M*	*SD*		*M*	*SD*	*M*	*SD*	
Depression symptoms	16.68	11.73	8.29	3.91	27.36	9.46	-30.75^***^	11.02	7.45	26.81	11.17	-18.55^***^
Depressed affect	3.95	4.81	0.79	1.18	7.97	4.69	-24.27^***^	1.69	2.78	8.00	5.01	-17.05^***^
Anhedonia	6.96	3.09	5.28	2.56	9.09	2.28	-19.23^***^	6.07	2.97	8.55	2.61	-10.25^***^
Somatic complaints	5.01	4.51	2.07	1.70	8.77	4.16	-24.59^***^	2.97	2.84	8.68	4.62	-16.48^***^
Interpersonal problems	0.76	1.26	0.15	0.43	1.54	1.51	-14.49^***^	0.30	0.75	1.58	1.54	-11.48^***^
Anxiety symptoms	4.11	4.57	1.71	2.69	7.17	4.64	-17.02^***^	1.25	1.28	9.24	3.80	-29.91^***^
Nervousness	0.58	0.79	0.22	0.50	1.05	0.86	-13.81^***^	0.14	0.35	1.38	0.74	-23.07^***^
Uncontrollable worry	0.62	0.81	0.25	0.50	1.08	0.90	-13.43^***^	0.20	0.40	1.37	0.83	-19.54^***^
Excessive worry	0.85	0.84	0.46	0.60	1.35	0.83	-14.59^***^	0.43	0.54	1.61	0.73	-20.72^***^
Trouble relaxing	0.59	0.76	0.24	0.52	1.04	0.79	-14.19^***^	0.18	0.40	1.32	0.71	-21.60^***^
Restlessness	0.42	0.73	0.13	0.45	0.78	0.86	-11.04^***^	0.05	0.22	1.07	0.86	-17.15^***^
Irritability	0.6	0.76	0.26	0.52	1.03	0.80	-13.47^***^	0.19	0.39	1.33	0.70	-22.09^***^
Feeling afraid	0.45	0.75	0.13	0.44	0.86	0.87	-12.38^***^	0.06	0.26	1.15	0.84	-18.51^***^
Physical factors												
Physical health	2.73	0.78	2.88	0.75	2.55	0.79	5.18^***^	2.85	0.73	2.52	0.83	4.90^***^
Number of chronic diseases	0.83	1.02	0.74	0.95	0.94	1.09	-2.34^*^	0.74	0.91	0.99	1.18	-2.90^**^
Sleep disturbance	7.37	3.58	6.14	2.86	8.95	3.78	-10.03^***^	6.32	3.01	9.27	3.75	-9.90^***^
Cognitive factors												
Perceived stress	16.28	5.44	13.35	4.43	20.02	4.17	-18.80^***^	13.90	4.45	20.54	4.36	-17.65^***^
Mastery	19.62	3.61	21.61	2.83	17.08	2.82	19.53^***^	20.88	3.28	17.35	3.02	13.00^***^
Social factors												
Social engagement	1.63	0.99	1.69	1.00	1.55	0.98	1.72	1.68	1.00	1.55	0.98	1.53
Social connection with family	1.99	0.91	2.17	0.90	1.76	0.87	5.69^***^	2.07	0.93	1.85	0.87	2.85^**^
Social connection with friend	1.74	0.91	1.87	0.88	1.57	0.92	3.97^***^	1.81	0.88	1.62	0.95	2.45^**^

^*^ *p*<.05. ^**^ *p*<.01. ^***^ *p*<.001.

### 2.3 Measures

All measures of depression and anxiety symptoms (39, 40) as well as physical, cognitive, and social factors that were used in this study have been validated in South Korean populations (41–43).

#### 2.3.1 Depression symptoms

Depression symptoms were assessed using the 20-item Center for Epidemiological Studies Depression Scale (CES-D) (44), which has four subscales of depressed affect, anhedonia, somatic complaints, and interpersonal problems. Detailed information of sample items is summarized in **Table 3**. Each item was scored from 0 (*not at all*) to 3 (*a lot*). The CES-D total scores range from 0 to 60. Total scores of 16 and 25 are considered the cutoffs for clinical and major depressive symptoms, respectively. The Cronbach’s α scores for depressed affect, anhedonia, somatic complaints, and interpersonal problems were 0.92, 0.85, 0.88, and 0.77, respectively.

**Table 3:** Item information of depression and anxiety scales.

Node	Item	Cronbach’s α
Depression symptoms (CES-D)		0.94
Depressed affect	I felt that I could not shake off the blues even with help from my family or friends.	0.92
	I felt depressed.	
	I thought my life had been a failure.	
	I felt fearful.	
	I felt lonely.	
	I had crying spells.	
	I felt sad.	
Anhedonia	I felt that I was just as good as other people.	0.85
	I felt hopeful about the future.	
	I was happy.	
	I enjoyed life.	
Somatic complaints	I was bothered by things that usually don’t bother me.	0.88
	I did not feel like eating; my appetite was poor.	
	I had trouble keeping my mind on what I was doing.	
	I felt that everything I did was an effort.	
	My sleep was restless.	
	I talked less than usual.	
	I could not get “going”.	
Interpersonal problems	People were unfriendly.	0.77
	I felt that people dislike me.	
Anxiety symptoms (GAD-7)		0.93
Nervousness	Feeling nervous, anxious, or on edge.	
Uncontrollable worry	Not being able to stop or control worrying.	
Excessive worry	Worrying too much about different things.	
Trouble relaxing	Trouble relaxing.	
Restlessness	Being so restless that it is hard to sit still.	
Irritability	Becoming easily annoyed or irritable.	
Feeling afraid	Feeling afraid, as if something awful might happen.	

#### 2.3.2 Anxiety symptoms

Anxiety symptoms were measured with the 7-item Generalized Anxiety Disorder Scale (GAD-7) (45). Detailed information of sample items is summarized in **Table 3**. Each item was scored from 0 (*not at all*) to 3 (*nearly every day*). The GAD-7 total scores range from 0 to 21. Total scores of 5, 10, 15 are considered the cut-offs for mild, moderate, and severe anxiety symptoms, respectively. The Cronbach’s α of this scale for this study was 0.93.

#### 2.3.3 Physical factors

Physical health was measured using a single item assessing perceived health (46) that is rated on a scale ranging from 1 (*poor*) to 5 (*excellent*). Chronic diseases were measured using an index of 13 major chronic diseases (e.g., cancer, respiratory diseases, and vascular diseases) by summing the number of diagnosed diseases. Sleep disturbance was measured using the 19-item Pittsburgh Sleep Quality Index (PSQI) (47), which assesses the quality and patterns of sleep (i.e., sleep quality, sleep latency, sleep duration, sleep efficiency, sleep disturbances, use of sleep medication, and daytime dysfunction) over a one-month duration. Sample items are “During the past month, how often have you taken medicine to help you sleep?” and “During the past month, how would you rate your sleep quality overall?” Each item was rated on a scale ranging from 1 (*very good*) to 4(*very bad*). PSQI total scores range from 0 to 21, with a higher score indicating greater sleep disturbance.

#### 2.3.4 Cognitive factors

Perceived stress was measured using the 10-item Perceived Stress Scale (PSS) (48), which assesses the extent to which situations in one’s life are perceived as stressful. A sample item is “How often have you been upset because of something that happened unexpectedly?” Each item was rated on a scale ranging from 0 (*never*) to 4 (*very often*). PSS scores range from 0 to 40, with a higher score indicating greater stress. The Cronbach’s α of this scale for this study was 0.78. Mastery (i.e., sense of control) was measured using the 7-item Pearlin Mastery Scale (PMS) (49), which assesses the extent to which individuals regard their life circumstances as being under control. A sample item is “What happens to me in the future mostly depends on me.” Each item was rated on a scale ranging from 1 (*strongly disagree*) to 4 (*strongly agree*). PMS total scores range from 7 to 28, with a higher score indicating greater levels of mastery. The Cronbach’s α of this scale for this study was 0.79.

#### 2.3.5 Social factors

Social connection and social engagement were measured using the Lubben Social Network Scale-18 (LSNS-18) (50, 51), which assesses the degree of social connection (i.e., network size, closeness, and frequency of contact) and social engagement (e.g., volunteer, religious, and leisure/culture/sport). Social connection consists of 6-item for each social partner, with a higher score indicating larger networks and more frequent social contact. Social engagement includes an index of seven social activities and the number of social activities was used to indicate the level of social engagement.

### 2.4 Statistical analyses

We estimated the network structure of depression and anxiety symptoms as well as physical, cognitive, and social factors. Physical factors included physical health, chronic diseases, and sleep disturbance, while cognitive factors included perceived stress and mastery (i.e., sense of control), and social factors included social connection and social engagement. The network structure was estimated based on the total sample first, and then potential gender differences were examined in relation to the overall network structure as well as item-level associations.

#### 2.4.1 Network estimation

All statistical analyses were conducted using R version 4.3.1. To check item redundancy prior to network estimation, *goldbricker* function in the R-package *networktools* version 1.5.2 was used. To estimate the network structures of depression and anxiety symptoms, as well as physical, cognitive, and social factors, we used the Extended Bayesian Information Criterion (EBIC) graphical least absolute shrinkage and selection operator (GLASSO) network model (52). To estimate and visualize the networks, we used the R-package *qgraph* version 1.9.8 (53). The nodes represented depression and anxiety symptoms and physical, cognitive, and social factors, while the edges represented the regularized partial correlation coefficients between the nodes, which excluded the multicollinearity of node correlations (54). A regularization algorithm was used to reduce all of the insignificant edges in the network to zero, which led to a sparse network with as few edges as possible (55). Thicker edges indicate higher correlations, while blue and red color edges indicate positive and negative correlations, respectively. Highly correlated nodes were placed closer together, while weakly correlated nodes were placed in the peripheral areas of the network graph (56).

#### 2.4.2 Network centrality

To assess the prominence of each node in the network, we estimated the centrality index of Strength (i.e., the sum of the absolute edge weights connected to a node) with higher strength denoting a more central role in the network (57). To detect bridge nodes that play an influential role in connecting two or more clusters, we estimated the bridge centrality index of Bridge Strength (i.e., the sum of the edge weights connecting a node to the nodes in other clusters). We used the R-package *bootnet* version 1.5.6 (58) and *networktools* version 1.5.1 (59) to compute both indices. Following recommendations from prior literature (60), the top 25% scoring nodes on the centrality indices were selected in the networks.

#### 2.4.3 Network accuracy and stability

The robustness of the networks was assessed using R-package *bootnet* version 1.5.6. We utilized a non-parametric bootstrapping procedure to estimate the accuracy of edge weights by calculating 95% confidence intervals (CIs), with a narrow CI indicating a reliable network. In addition, we used a case-dropping bootstrap procedure to assess the network stability by calculating the correlation stability-coefficients (CS-coefficients). CS-coefficient values above 0.25 are recommended for interpretation (61).

#### 2.4.4 Network comparison

To assess potential gender differences in the overall network structure, global strength, and edge strength, the R-package *Network Comparison Test* was used (62), utilizing the Benjamini and Hochberg false discovery rate correction to control the family-wise error rate.

## 3 Results

### 3.1 Network structure

**Figure 1:**
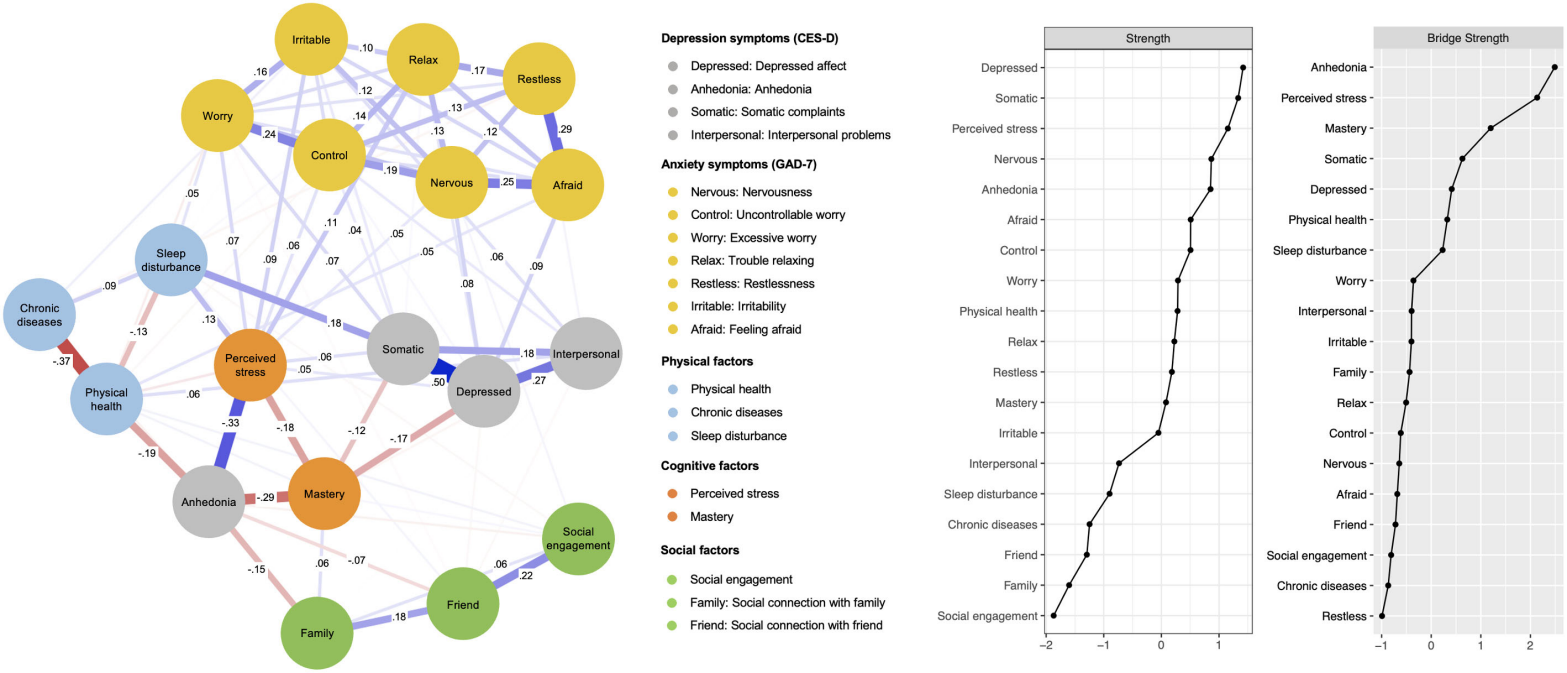
The network structure of depression and anxiety symptoms and physical, cognitive, and social factors.

As presented in **Figure 1**, the network structure of depression and anxiety symptoms and physical, cognitive, and social factors indicated that 83 of 171 edges (48.54%) were above zero. As there were no redundant items, all items of physical, cognitive, and social domains were included in the network model. With regard to the depression symptoms, depressed affect and somatic complaints displayed the strongest connection, followed by the edge for depressed affect and interpersonal problems. Among the anxiety symptoms, feeling afraid and restlessness showed the strongest connection, followed by the edge for feeling afraid and nervousness. Between depression and anxiety symptoms, depressed affect and feeling afraid, and depressed affect and nervousness displayed strong connections. With regard to the link between depression and anxiety symptoms and physical and cognitive factors, perceived stress weighed the highest in connections with both depression and anxiety symptoms, followed by mastery, sleep disturbance, and physical health, suggesting their substantial impact on depression and anxiety. Perceived stress was positively associated with most of the depression and anxiety symptoms and had particularly strong connection with anhedonia. In contrast, mastery was negatively associated with both perceived stress and depression symptoms and had a particularly strong connection with anhedonia. Sleep disturbance had positive connections with depression and anxiety symptoms and physical health was negatively associated with anhedonia. With regard to the social factors, although the relative strength of their impact on depression and anxiety symptoms was weaker than those of physical and cognitive factors, social connection with family was negatively associated with anhedonia. It is noteworthy that social engagement had strong positive connection with friends. The weights matrix for the regularized partial correlation network estimation is provided in **Supplementary Table S2**. The predictability index showed that 45.18% of the variance in each node was explained by the adjacent nodes. **Supplementary Figures S1, S2** provides the results of the bootstrapped 95% CIs of edge weights and bootstrapped difference tests for edge weights.

### 3.2 Network centrality

According to the centrality index of Strength (see **Figure 1**), depressed affect had the highest strength, followed by somatic complaints, perceived stress, nervousness, anhedonia, feeling afraid, and uncontrollable worry, indicating these nodes were the most strongly associated other symptoms or factors in the network. The top 25% of scoring nodes on Bridge Strength are also displayed in **Figure 1**. Anhedonia emerged as the most central bridge node, followed by perceived stress, and mastery, indicating these were the main bridge nodes connecting depression and anxiety symptoms and relevant risk and resilience factors. Perceived stress acted as a strong bridge node in connecting most depression and anxiety symptoms. Anhedonia served as a strong bridge node in connecting perceived stress, mastery, physical health, and social connection with family. Mastery acted as a strong bridge node in connecting perceived stress, anhedonia, and depressed affect as well as somatic complaints. **Supplementary Figure S3** provides the results of the non-parametric bootstrapped difference tests of Strength.

### 3.3 Network stability

Regarding network stability, the case dropping bootstrap procedure showed that Strength and Bridge Strength values remained stable after dropping different proportions of the sample (see **Supplementary Figure S4**). The CS-coefficients for Strength and Bridge Strength were both 0.75, indicating that the results did not change significantly after dropping 75% of the sample.

### 3.4 Network comparison

**Figure 2:**
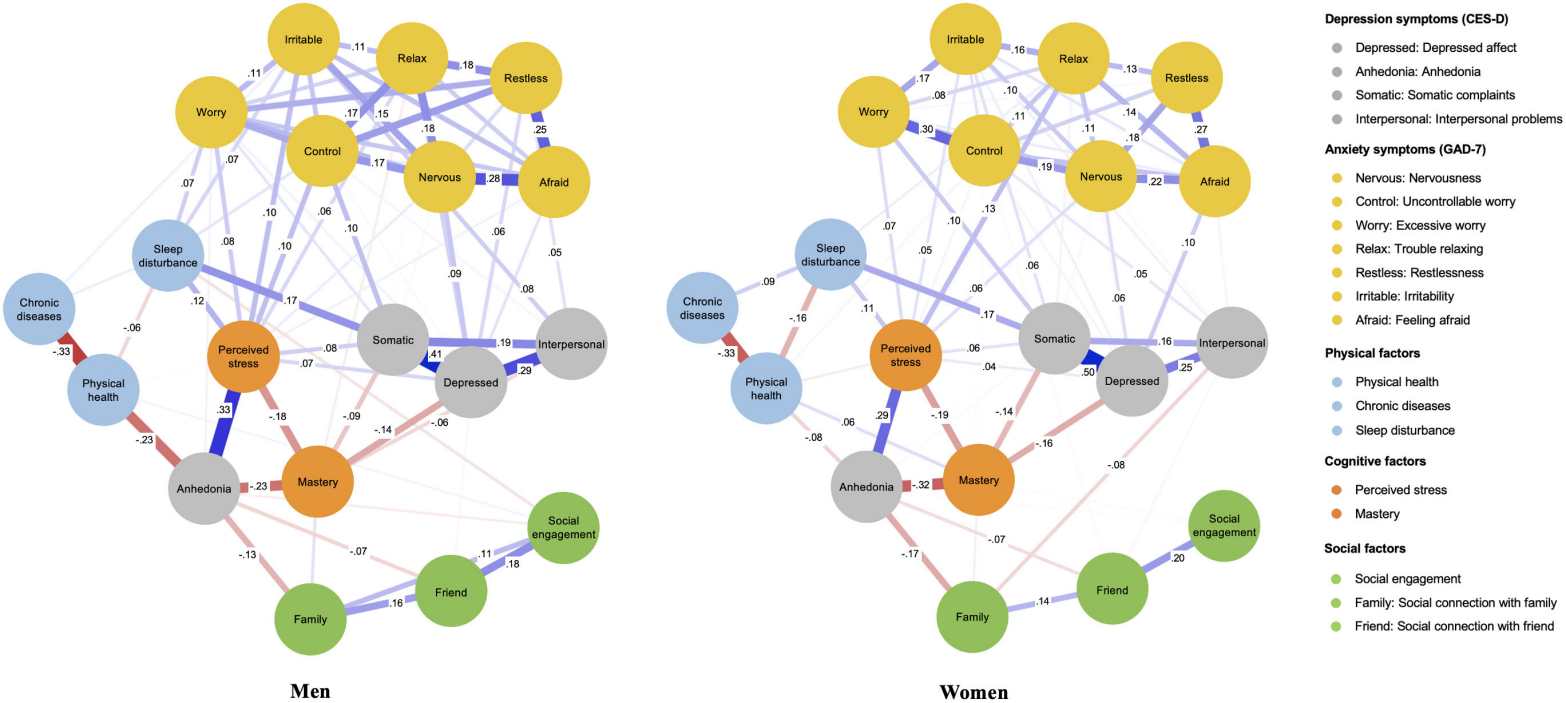
The network structure of estimated models for middle-aged men and women.

**Figure 3:**
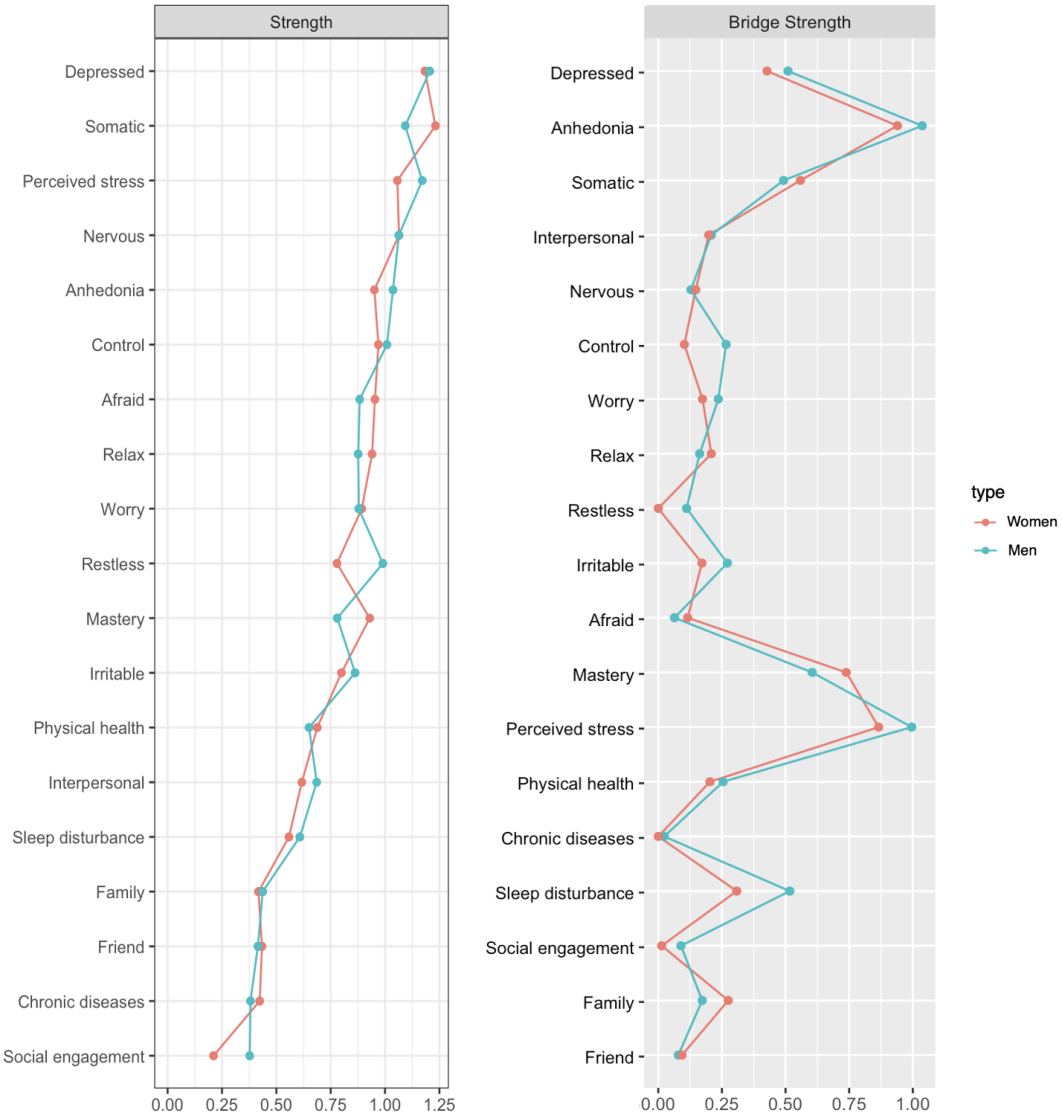
Centrality indices of the estimated models for middle-aged men and women.

The overall network structure was not significantly different between men and women (maximum difference = 0.17; *p* = 0.73) and the network comparison test showed no significant difference in global strengths (global strength difference = 0.15; global men strength = 7.70; global women strength = 7.55; *p* = 0.64). However, after the Benjamini and Hochberg *post hoc* comparison correction, we found six edges that were significantly different between men and women; Uncontrollable worry–excessive worry, and interpersonal problems–social connection with family had significantly stronger edges in women than in men, whereas somatic complaints–uncontrollable worry, depressed affect–restlessness, anhedonia–physical health, irritability–sleep disturbance had significantly stronger edges in men than in women. The estimated network structures for men and women are presented in **Figure 2** and centrality indices are shown in **Figure 3**. CS-coefficients for Strength and Bridge Strength were both 0.75 in the network of men and women. The weights matrix for the regularized partial correlation network estimation by gender is provided in **Supplementary Table S3**. The CS-coefficient graphs for the total sample and for men and women are provided in **Supplementary Figures S5–S8**. For the centrality index of Strength and Bridge Strength, perceived stress was the most central factor and perceived stress and mastery were the main bridge factors for depression and anxiety symptoms in both men’s and women’s networks.

## 4 Discussion

This study used network analysis to characterize the network structure of depression and anxiety symptoms and physical, cognitive, and social factors among middle-aged men and women. With this approach, we quantified the complex and interactive relationships within and between depression and anxiety symptoms and various risk and resilience factors, and identified multiple pathways linking depression and anxiety symptoms and individual and environmental factors. The network of depression and anxiety symptoms, the associations between depression and anxiety symptoms and risk and resilience factors, and gender differences in the strength of these associations are discussed in more detail below.

Network analysis results revealed that depressed affect and somatic complaints of depression symptoms, and nervousness and feeling afraid of anxiety symptoms were central symptoms, indicating that these symptoms have the strongest impact in maintaining the entire symptom network. These findings are consistent with current understanding of the primary symptoms required for a diagnosis of major depression and anxiety disorders (63) as well as previous network analyses results (64, 65). Moreover, anhedonia was found to be the central bridge symptom in the network, reflecting reductions in interest and pleasure function as a major pathway between depression and anxiety. Central and bridge symptoms identified in this study have been consistently understood as core symptoms of depression and anxiety in both psychiatric and non-psychiatric samples (63, 66). Our findings corroborate the results obtained in previous studies and underscore that depressed affect, somatic complaints, and anhedonia, as well as nervousness and feeling afraid can be considered central hallmark symptoms of affective disorders that are the most likely candidates for triggering or activating remaining depression and anxiety symptoms.

With regard to the link between depression and anxiety symptoms and physical, cognitive, and social factors, perceived stress was the highest risk factor, while mastery was the strongest resilience factor. These two had the most prominent roles for depression and anxiety symptoms. Although perceived stress was positively associated with most of the depression and anxiety symptoms, it had a particularly strong connection with anhedonia. Moreover, anhedonia and perceived stress were the most central bridge factors in the entire network. The strength of anhedonia and perceived stress and their bridging roles in the network indicate the importance of perception of stress and loss of interest and pleasure as central connections between various depression and anxiety symptoms and relevant factors. Prior research has provided strong evidence that chronic stress contributes to the emergence of anhedonia (26, 67) and is a strong predictor of development and maintenance of psychopathology symptoms (68). The current results further emphasize that the impact of perceived stress on depression and anxiety is more substantial relative to other individual and environmental factors, and perceived stress and anhedonia can play a key role in the onset and development of depression and anxiety symptoms and can exacerbate the comorbidity and severity of depression and anxiety by functioning as important pathways and shared underlying causes.

It is noteworthy that perceived stress, anhedonia, and mastery were tightly clustered together and exhibit strong inter-connections to each other. Mastery was negatively associated with both perceived stress and depression symptoms and had particularly strong and negative connections with anhedonia. Moreover, mastery was another central bridge factor following anhedonia and perceived stress in the entire network. Overall, stress, anhedonia, and mastery within the network were clearly key components within which other clusters were connected. The finding that mastery served a central bridging role indicates that beliefs of control over life can be protective from developing depression and anxiety symptoms in the face of stressful events. Maladaptive cognitions such as believing one has a lack of control have been considered important determinants for endorsement of depression and anxiety symptoms (69). Perceptions of lack of control can lead to certain cognitive patterns dominated by worry, fixation on threat, rumination, and other counter-productive thought processes, and often results in conditions where negative emotions persist (29, 70). Abundant research has revealed that individuals with depression and anxiety symptoms often display avoidant tendencies or overwhelmed emotional experiences, which further contributes to depressed affective states and increased worry, in a fashion which is cyclical in nature (71). The densely interconnected cluster of stress, anhedonia, and mastery further substantiate the important connections between perceptions of stress, (mal)adaptive cognitions, and depression and anxiety, such that mastery or a sense of control may be associated with more positive emotions, thus buffering against the negative impact of stress (28, 72).

Although overall network structure was not significantly different, there were several associations that differed in important ways between men and women. The connections of physical health-anhedonia and sleep disturbance-irritability were stronger in men than in women, while social connection with family was linked to interpersonal problems only in women. These results can be diagnostically useful as physical health and sleep disturbances are more critically related to depression and anxiety symptoms for men, whereas social factors are more influential for depression and anxiety symptoms in women. Previous research has provided strong evidence that physical functioning or illness contribute to psychiatric conditions where affective disorders are centrally relevant (e.g., anhedonia) (24). Abundant research has also indicated that sleep disturbances such as insomnia contribute to the onset and recurrence of internalizing symptoms (22, 23, 73). However, evidence of gender differences in terms of impact of physical health and sleep disturbances on depression and anxiety is scarce and mixed. Several studies have reported that men with sleep disorders reported higher levels of fatigue and depression symptoms compared with women (74, 75), but other studies have also indicated physical activity was more effective in alleviating depression symptoms in women than in men (76, 77). Given that these studies have used aggregated scores of depression symptoms, future studies based on symptom-level interpretations should analyze the link between physical factors and depression symptoms to check how generalizable the current findings are.

In contrast, gender-related differences in terms of the links between social connection and depression symptoms have been supported in the literature and are consistent with the current findings. Multiple studies have reported that although women can benefit more from supportive relationships than men, they can also feel increased stress from the additional roles they are expected to play and their involvement in the lives of others (78). Moreover, women can suffer more when they have conflict with their social partners or experience a lack of support, due to high expectations and devotion to social connection (79). Previous studies have consistently shown that women feel more responsible toward social partners than men and this can cause women to feel more burdened and overloaded (80). Greater involvement and responsibility have been associated with higher rates of depression in women than in men (81, 82). The negative link between social connection with anhedonia as well as perceived stress was robust and equally strong for both men and women, corroborating the protective benefits of social connection for depression and anxiety symptoms (33, 34). However, potential gender differences found in the present study underscore that healthcare providers should aim to provide more support that is tailored to gender.

The current findings have several clinical implications. In view of the central impact of bridge nodes in the network, prevention and intervention efforts should target central bridge symptoms or factors that serve as key pathways for co-occurring depression and anxiety. Anhedonia, which is characterized by loss of motivation, interest, and pleasure emerged as the most prominent bridge symptom in the current comorbidity network. Considering its dense interconnection with mastery and perceived stress, anhedonia may connect transdiagnostic experiences between depression and anxiety, such as avoidance tendency or overwhelmed emotions, and reduced interest (83). Hence, targeting anhedonia would be beneficial to prevent or alleviate depression and anxiety symptoms among middle-aged men and women. In a related vein, the findings that mastery served as central bridging roles and had strong and negative connections with perceived stress and anhedonia suggests that one’s belief about control over stressful events in life can be protective from the onset of affective disorders. Accordingly, mental health preventions should focus on building mastery or enhancing coping strategies to foster a sense of control in the face of stressful events through a treatment program. Lastly, our results showed that the connections of physical health–anhedonia and sleep disturbance–irritability were stronger in men than in women. Engaging in behavioral activities to maintain physical health (e.g., exercise and taking a walk), ensuring adequate sleep, and other self-care activities may therefore be particularly beneficial in reducing overall symptom severity for men.

There are a number of limitations. First, this study was based on a cross-sectional analysis, which is not sufficient to make causal inferences between symptoms and risk and resilience factors. Future study should clarify how physical, cognitive, and social factors interact with each other and predict symptom-level changes by employing longitudinal network analysis. Second, although considering multiple domains of physical, cognitive, and social factors and analyzing their interrelationships with depression and anxiety symptoms is a strength of this study, there are other important factors that were not included. Future studies could take into account a more diverse range of individual and environmental factors and thus provide a more comprehensive portrait of the complex pattern of interplay between risk and resilience factors and psychopathology symptoms. Third, although the current sample is deemed to be representative of South Korean middle-aged adult population with the use of a stratified sampling, the modest sample size may limit the generalizability of the findings. Future research based on different cohorts should replicate our results to allow for the generalization of the interrelationships found in this study.

## 5 Conclusion

This study is the first to consider various risk and resilience factors in physical, cognitive, and social domains and simultaneously investigate their interrelationships with depression and anxiety symptoms among middle-aged men and women, by employing a network analysis. Through this approach, we have elucidated the complex associations within and between physical, cognitive, social, and psychological domains, and uncover various pathways linking risk and resilience factors with comorbid depression and anxiety symptoms. Identifying the protective or harmful roles of individual and environmental factors on depression and anxiety will be particularly informative given the enormous burden of depression and anxiety during mid-adulthood. The findings emphasize that prevention aimed at reducing perceptions of stress and promoting mastery, is central to preventing the onset or maintenance of depression and anxiety symptoms among middle-aged men and women.

## Data availability statement

The original contributions presented in the study are included in the article/**Supplementary Material**. Further inquiries can be directed to the corresponding author.

## Ethics statement

The studies involving humans were approved by Institutional Review Board, Jeonbuk National University. The studies were conducted in accordance with the local legislation and institutional requirements. The participants provided their written informed consent to participate in this study.

## Author contributions

HS: Validation, Supervision, Project administration, Methodology, Investigation, Data curation, Conceptualization, Writing – review & editing, Writing – original draft. CP: Writing – review & editing, Formal Analysis, Data curation.
