# Supplementary material for: Mastery is central: an examination of complex interrelationships between physical health, stress and adaptive cognition, and social connection with depression and anxiety symptoms
Source: Front Psychiatry. 2024 May 1;15:1401142. doi: 10.3389/fpsyt.2024.1401142 (PMC11094708; doi:10.3389/fpsyt.2024.1401142)
Supplement: Supplementary file 1 [file DataSheet_1.docx]

**Supplementary Materials**

Table S1

*Descriptive information of gender differences*

|  | Male (*n* = 289) | |  | Female (*n* = 311) | | *t* | *p*-value | 95% of the confidence interval |
| --- | --- | --- | --- | --- | --- | --- | --- | --- |
| Variable | *M* | *SD* |  | *M* | *SD* |  |  |  |
| Depression symptoms | 16.55 | 11.62 |  | 16.80 | 11.86 | -0.25 | 0.80 | [-2.13, 1.64] |
| Depressed affect | 3.87 | 4.62 |  | 4.02 | 4.99 | -0.39 | 0.70 | [-0.93, 0.62] |
| Anhedonia | 6.75 | 3.04 |  | 7.15 | 3.12 | -1.62 | 0.11 | [-0.90, 0.09] |
| Somatic complaints | 5.01 | 4.54 |  | 5.02 | 4.48 | -0.02 | 0.99 | [-0.73, 0.72] |
| **Interpersonal problems** | **0.93** | **1.40** |  | **0.60** | **1.09** | **3.14^**^** | **< 0.01** | **[ 0.12, 0.52]** |
| Anxiety symptoms | 4.04 | 4.55 |  | 4.18 | 4.59 | -0.35 | 0.72 | [-0.87, 0.60] |
| Nervousness | 0.55 | 0.73 |  | 0.62 | 0.85 | -1.04 | 0.30 | [-0.19, 0.06] |
| Uncontrollable worry | 0.61 | 0.79 |  | 0.63 | 0.84 | -0.32 | 0.75 | [-0.15, 0.11] |
| **Excessive worry** | **0.79** | **0.80** |  | **0.92** | **0.86** | **-1.87^†^** | **0.06** | **[-0.26, 0.01]** |
| Trouble relaxing | 0.60 | 0.78 |  | 0.59 | 0.75 | 0.16 | 0.87 | [-0.11, 0.13] |
| Restlessness | 0.45 | 0.74 |  | 0.39 | 0.72 | 0.96 | 0.34 | [-0.06, 0.17] |
| Irritability | 0.59 | 0.74 |  | 0.61 | 0.77 | -0.37 | 0.71 | [-0.14, 0.10] |
| Feeling afraid | 0.47 | 0.77 |  | 0.43 | 0.74 | 0.64 | 0.52 | [-0.08, 0.16] |
| Physical factors |  |  |  |  |  |  |  |  |
| **Physical health** | **2.80** | **0.78** |  | **2.68** | **0.78** | **1.89^†^** | **0.06** | **[ 0.01, 0.25]** |
| Number of chronic diseases | 0.84 | 1.00 |  | 0.82 | 1.04 | 0.17 | 0.87 | [-0.15, 0.18] |
| Sleep disturbance | 7.14 | 3.46 |  | 7.59 | 3.68 | -1.53 | 0.13 | [-1.02, 0.13] |
| Cognitive factors |  |  |  |  |  |  |  |  |
| Perceived stress | 15.97 | 5.46 |  | 16.58 | 5.41 | -1.37 | 0.17 | [-1.48, 0.26] |
| Mastery | 19.63 | 3.57 |  | 19.60 | 3.65 | 0.10 | 0.92 | [-0.55, 0.61] |
| Social factors |  |  |  |  |  |  |  |  |
| Social engagement | 1.63 | 0.92 |  | 1.64 | 1.06 | -0.13 | 0.90 | [-0.17, 0.15] |
| **Social connection with family** | **1.86** | **0.85** |  | **2.11** | **0.95** | **-3.49^***^** | **< 0.001** | **[-0.40, -0.11]** |
| Social connection with friend | 1.69 | 0.90 |  | 1.78 | 0.92 | -1.24 | 0.22 | [-0.24, 0.05] |

^†^ *p* < .10. ^*^*p* < .05. ^**^*p* < .01. ^***^*p* < .001.

Table S2

*Weighted matrix for the regularized partial correlation network estimation*

|  | 1 | 2 | 3 | 4 | 5 | 6 | 7 | 8 | 9 | 10 | 11 | 12 | 13 | 14 | 15 | 16 | 17 | 18 | 19 |
| --- | --- | --- | --- | --- | --- | --- | --- | --- | --- | --- | --- | --- | --- | --- | --- | --- | --- | --- | --- |
| 1. Depressed | – |  |  |  |  |  |  |  |  |  |  |  |  |  |  |  |  |  |  |
| 2. Anhedonia | .00 | – |  |  |  |  |  |  |  |  |  |  |  |  |  |  |  |  |  |
| 3. Somatic | **.50** | .00 | – |  |  |  |  |  |  |  |  |  |  |  |  |  |  |  |  |
| 4. Interpersonal | **.27** | .00 | **.18** | – |  |  |  |  |  |  |  |  |  |  |  |  |  |  |  |
| 5. Nervous | **.08** | .00 | .00 | .00 | – |  |  |  |  |  |  |  |  |  |  |  |  |  |  |
| 6. Control | **.01** | .00 | **.04** | **.04** | **.19** | – |  |  |  |  |  |  |  |  |  |  |  |  |  |
| 7. Worry | .00 | .00 | **.07** | .00 | **.12** | **.24** | – |  |  |  |  |  |  |  |  |  |  |  |  |
| 8. Relax | **.05** | .00 | **.01** | .00 | **.13** | **.14** | **.07** | – |  |  |  |  |  |  |  |  |  |  |  |
| 9. Restless | .00 | .00 | .00 | **.02** | **.12** | **.13** | **.06** | **.17** | – |  |  |  |  |  |  |  |  |  |  |
| 10. Irritable | .00 | .00 | .**04** | **.06** | **.12** | **.07** | **.16** | **.10** | **.10** | – |  |  |  |  |  |  |  |  |  |
| 11. Afraid | **.09** | .00 | .00 | .00 | **.25** | **.07** | **.06** | **.11** | **.29** | **.08** | – |  |  |  |  |  |  |  |  |
| 12. Physical health | .00 | **-.19** | .00 | **.06** | .00 | .00 | **-.03** | **-.01** | .00 | .00 | **.05** | – |  |  |  |  |  |  |  |
| 13. Chronic diseases | .00 | **-.03** | .00 | .00 | .00 | .00 | .00 | .00 | **-.02** | **.03** | .00 | **-.37** | – |  |  |  |  |  |  |
| 14. Sleep disturbance | .00 | .00 | **.18** | .00 | .00 | .00 | **.05** | **.01** | .00 | .00 | .00 | **-.13** | **.09** | – |  |  |  |  |  |
| 15. Perceived stress | **.05** | **.33** | **.06** | .00 | **.05** | **.06** | **.07** | **.11** | .00 | **.09** | .00 | **-.04** | .00 | **.13** | – |  |  |  |  |
| 16. Mastery | **-.17** | **-.29** | **-.12** | **-.02** | .00 | .00 | .00 | .00 | .00 | .00 | .00 | **.02** | .00 | .00 | **-.18** | – |  |  |  |
| 17. Social engagement | .00 | **-.03** | .00 | .00 | **.02** | .00 | .00 | .00 | .00 | .00 | .00 | **.02** | .00 | **-.01** | .00 | **.02** | – |  |  |
| 18. Family | .00 | **-.15** | .00 | .00 | .00 | .00 | .00 | .00 | .00 | .00 | .00 | .00 | .00 | .00 | .00 | **.06** | **.06** | – |  |
| 19. Friend | **-.02** | **-.07** | .00 | **-.01** | .00 | .00 | **-.01** | .00 | .00 | .00 | .00 | .00 | .00 | **.02** | .00 | **.00** | **.22** | **.18** | – |

*‘*

Table S3

*Weighted matrix for the regularized partial correlation network estimation by gender*

|  | 1 | 2 | 3 | 4 | 5 | 6 | 7 | 8 | 9 | 10 | 11 | 12 | 13 | 14 | 15 | 16 | 17 | 18 | 19 |
| --- | --- | --- | --- | --- | --- | --- | --- | --- | --- | --- | --- | --- | --- | --- | --- | --- | --- | --- | --- |
| 1. Depressed | – | .00 | **.50** | **.25** | **.06** | **.02** | .00 | **.01** | .00 | **.03** | **.10** | .00 | .00 | .00 | **.04** | **-.16** | .00 | .00 | .00 |
| 2. Anhedonia | .00 | – | **.01** | .00 | .00 | .00 | .00 | .00 | .00 | .00 | .00 | **-.08** | .00 | .00 | **.29** | **-.32** | **-.01** | **-.17** | **-.07** |
| 3. Somatic | **.41** | .00 | – | **.16** | **.01** | .00 | **.10** | **.02** | .00 | **.06** | .00 | .00 | .00 | **.17** | **.06** | **-.14** | .00 | .00 | .00 |
| 4. Interpersonal | **.29** | .00 | **.19** | – | **.01** | **.05** | .00 | .00 | .00 | **.03** | **.02** | .00 | .00 | .00 | .00 | .00 | .00 | **-.08** | **-.02** |
| 5. Nervous | **.09** | .00 | **.01** | .00 | – | **.19** | **.11** | **.11** | **.18** | **.10** | **.22** | .00 | .00 | .00 | **.06** | .00 | .00 | .00 | .00 |
| 6. Control | **.01** | .00 | **.10** | **.01** | **.17** | – | **.30** | **.11** | **.09** | **.05** | **.12** | .00 | .00 | .00 | **.02** | **-.01** | .00 | .00 | **-.01** |
| 7. Worry | **.02** | **.02** | **.04** | .00 | **.11** | **.13** | – | **.08** | **.02** | **.17** | **.04** | .00 | .00 | .00 | **.07** | .00 | .00 | .00 | .00 |
| 8. Relax | **.07** | .00 | .00 | .00 | **.18** | **.17** | **.07** | – | **.13** | **.16** | **.14** | **-.02** | .00 | **.03** | **.13** | .00 | .00 | .00 | .00 |
| 9. Restless | **.06** | .00 | .00 | **.05** | **.06** | **.16** | **.13** | **.18** | – | **.09** | **.27** | .00 | .00 | .00 | .00 | .00 | .00 | .00 | .00 |
| 10. Irritable | .00 | .00 | .00 | **.08** | **.15** | **.09** | **.11** | **.04** | **.11** | – | **.05** | .00 | .00 | .00 | **.05** | .00 | .00 | .00 | .00 |
| 11. Afraid | **.04** | .00 | .00 | .00 | **.28** | **.02** | **.09** | **.08** | **.25** | **.10** | – | .00 | .00 | .00 | .00 | .00 | .00 | .00 | .00 |
| 12. Physical health | .00 | **-.23** | .00 | .00 | .00 | .00 | .00 | .00 | .00 | .00 | .00 | – | **-.33** | **-.16** | **-.04** | **.06** | .00 | .00 | .00 |
| 13. Chronic diseases | .00 | .00 | .00 | .00 | .00 | .00 | .00 | .00 | .00 | **.02** | .00 | **-.33** | – | **.09** | .00 | .00 | .00 | .00 | .00 |
| 14. Sleep disturbance | .00 | .00 | **.17** | .00 | .00 | **.04** | **.07** | .00 | .00 | **.07** | .00 | **-.06** | **.03** | – | **.11** | .00 | .00 | .00 | .00 |
| 15. Perceived stress | **.07** | **.33** | **.08** | .00 | **.03** | **.10** | **.08** | **.06** | .00 | **.10** | **.02** | .00 | .00 | **.12** | – | **-.19** | .00 | .00 | .00 |
| 16. Mastery | **-.14** | **-.23** | **-.09** | **-.06** | .00 | .00 | .00 | **-.03** | .00 | .00 | .00 | .00 | .00 | .00 | **-.18** | – | **.01** | **.03** | .00 |
| 17. Social engagement | .00 | **-.03** | .00 | .00 | .00 | .00 | .00 | .00 | .00 | .00 | .00 | **.02** | .00 | **-.04** | .00 | .00 | – | .00 | **.20** |
| 18. Family | .00 | **-.13** | .00 | .00 | .00 | .00 | .00 | .00 | .00 | .00 | .00 | .00 | .00 | .00 | .00 | **.04** | **.11** | – | **.14** |
| 19. Friend | **-.01** | **-.07** | .00 | .00 | .00 | .00 | .00 | .00 | .00 | .00 | .00 | .00 | .00 | .00 | .00 | .00 | **.18** | .16 | – |

*Note.* The lower left value is the partial correlation coefficient of men, while the upper right values is that of women.


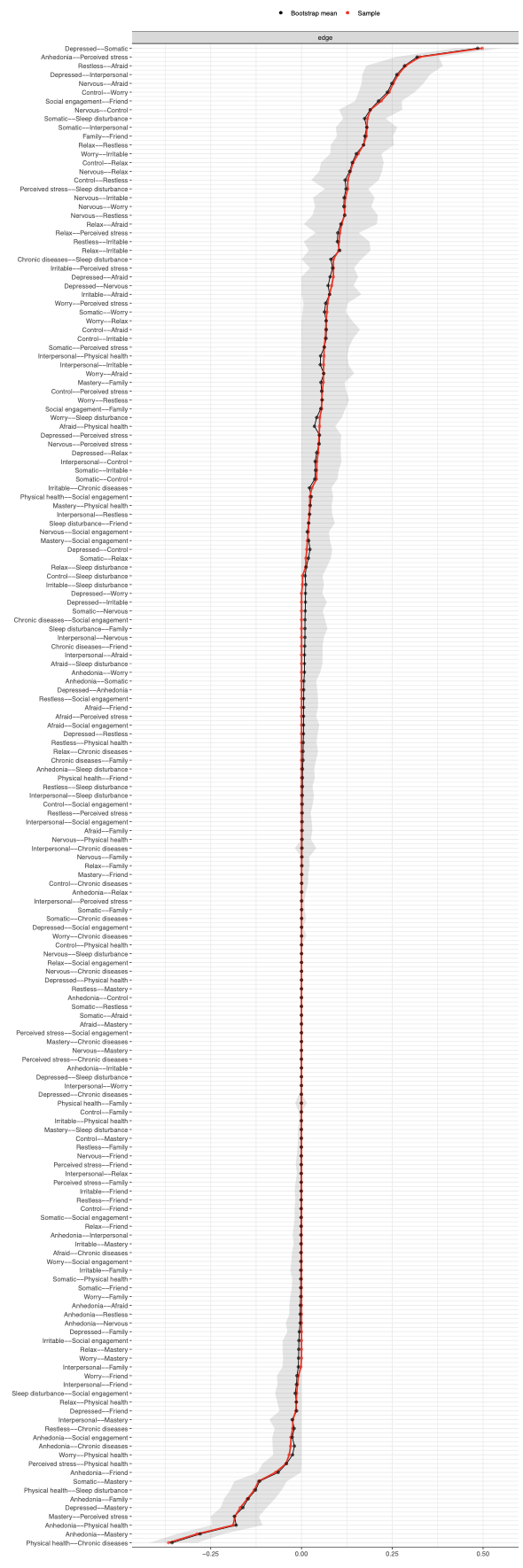


Figure S1

*Bootstrapped 95% confidence intervals of edge weights*

The black dots indicate the values of edge weight and the grey area indicates 95% confidence intervals of edge weights, estimated with the non-parametric bootstrap procedure. The sample values lie within relatively narrow CIs, indicating high stability.


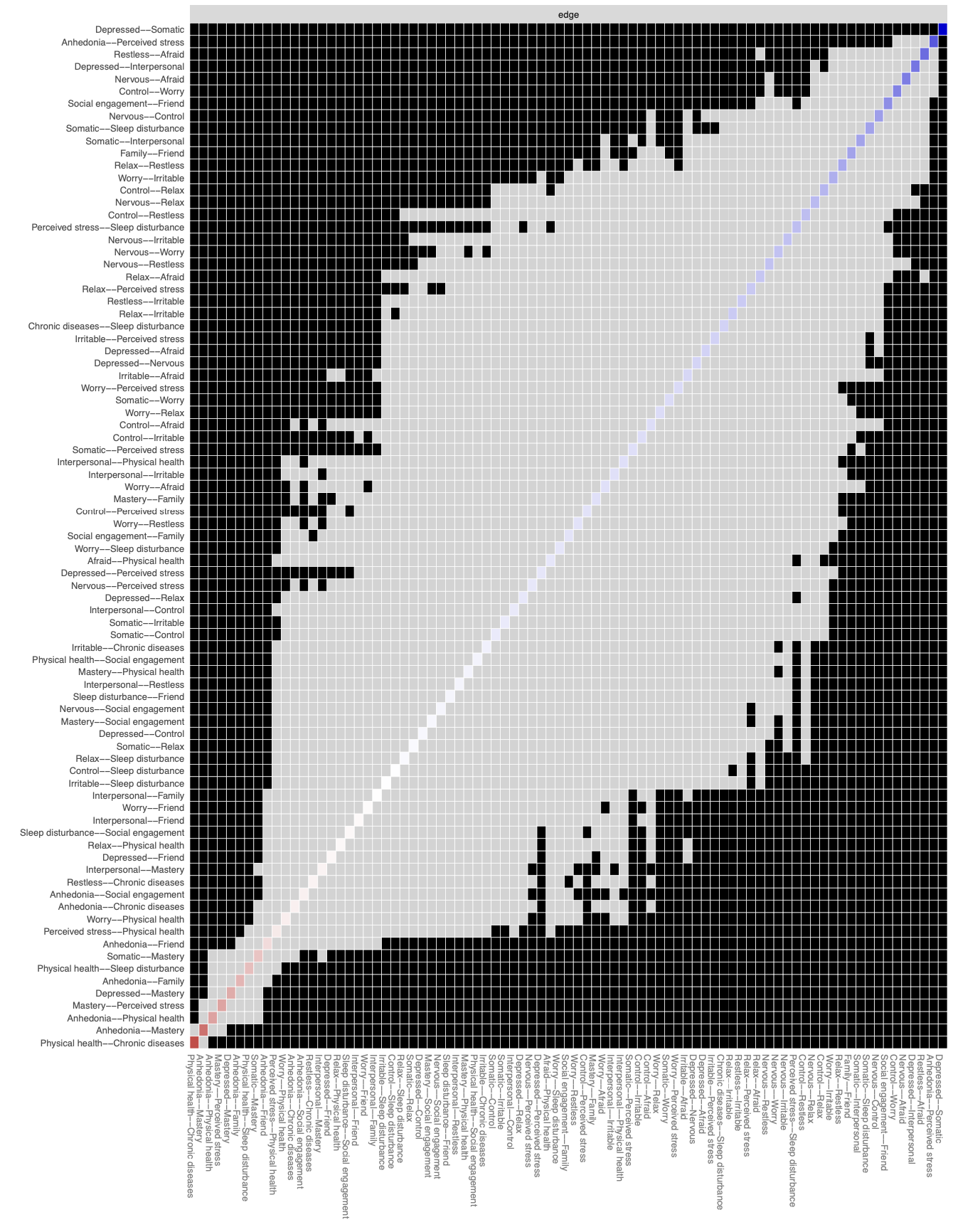


Figure S2

*Estimation of edge weight difference by bootstrapped difference test*

Bootstrapped difference tests between edge weights. Black boxes indicate significant differences (α = 0.05) between edge weights, whereas gray boxes indicate non-significant differences between edge weights. Blue boxes in the edge-weight plot indicate positive correlations.


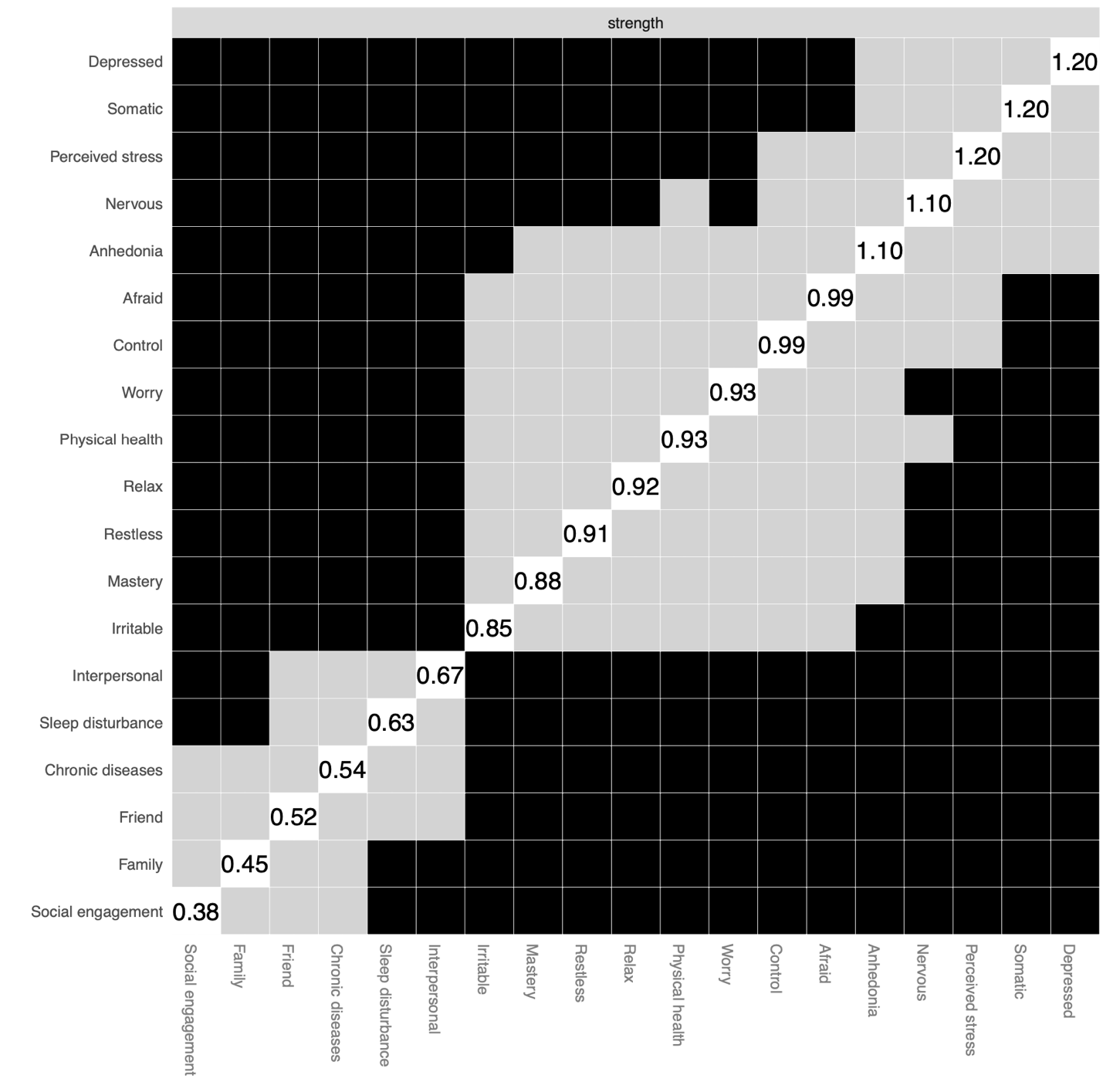


Figure S3

*Estimation of node strength difference by bootstrapped difference test*

Bootstrapped difference tests between node strength of depression and anxiety symptoms, and physical, cognitive, and social factors. Black boxes indicate nodes that are significantly different from one-another (α = 0.05), whereas gray boxes indicate non-significant differences. Numbers in the white boxes indicate the values of node strength.


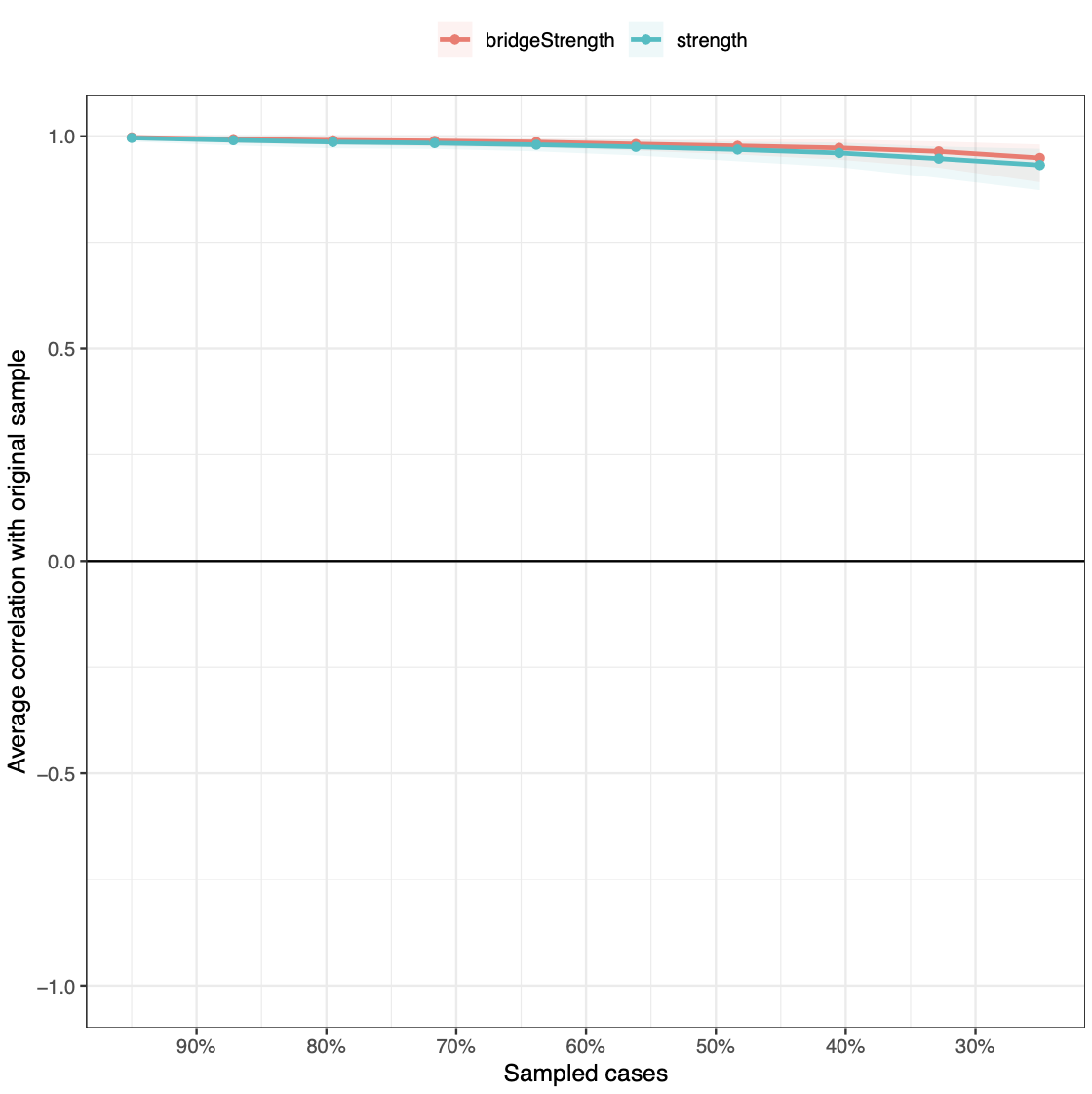


Figure S4

*Case-Dropping bootstrapped centrality indices*

Red and blue lines indicate mean correlations between centrality values of original and sub samples when dropping different proportions of the data; Straighter line indicates more reliable centrality. Areas around the lines indicate 95% CIs. The plot of the estimated networks indicates stable and reliable centrality.


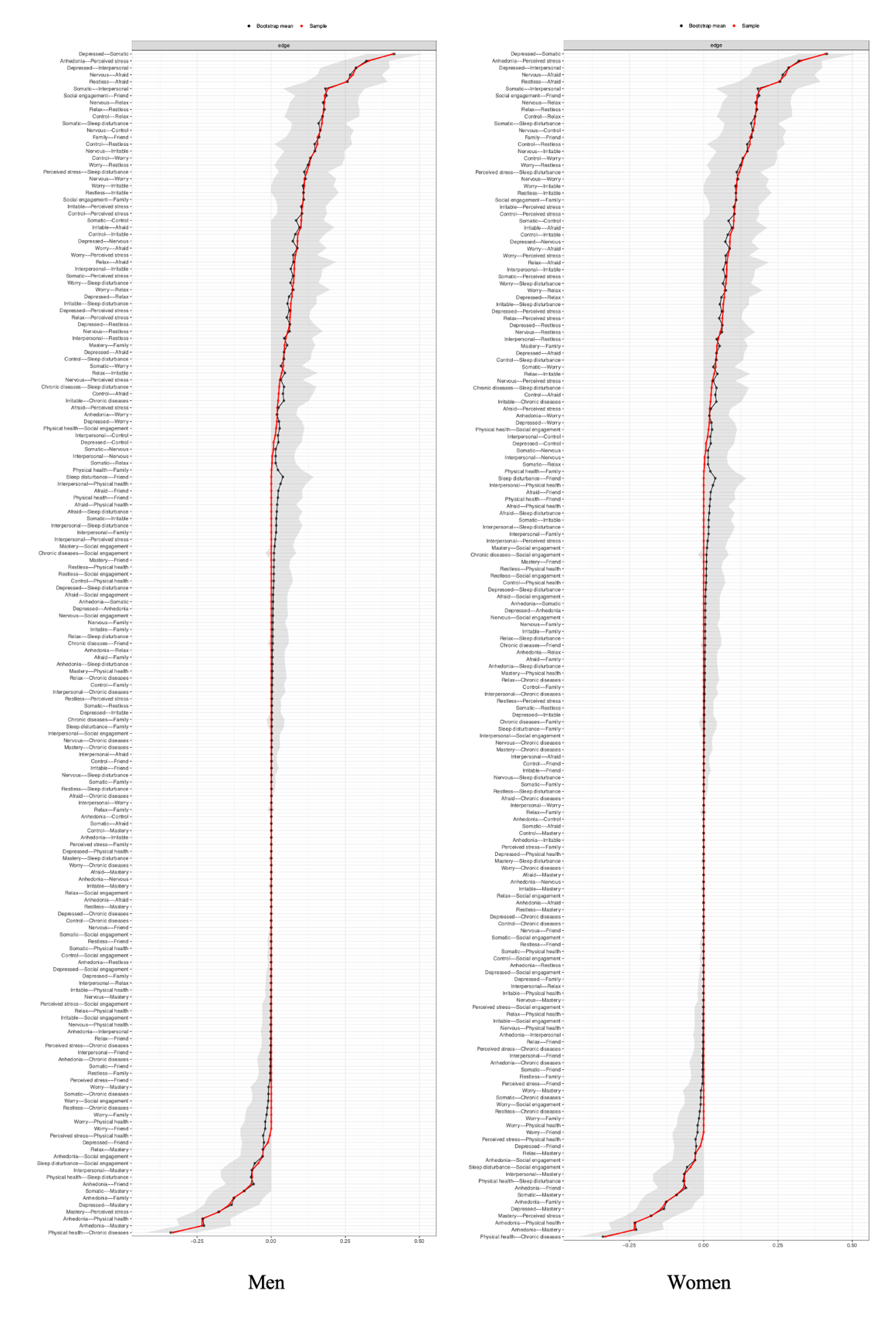


Figure S5

*Bootstrapped 95% confidence intervals of edge weights by gender*

The black dots indicate the values of edge weight and the grey area indicates 95% confidence intervals of edge weights, estimated with the non-parametric bootstrap procedure. The sample values lie within relatively narrow CIs, indicating high stability.


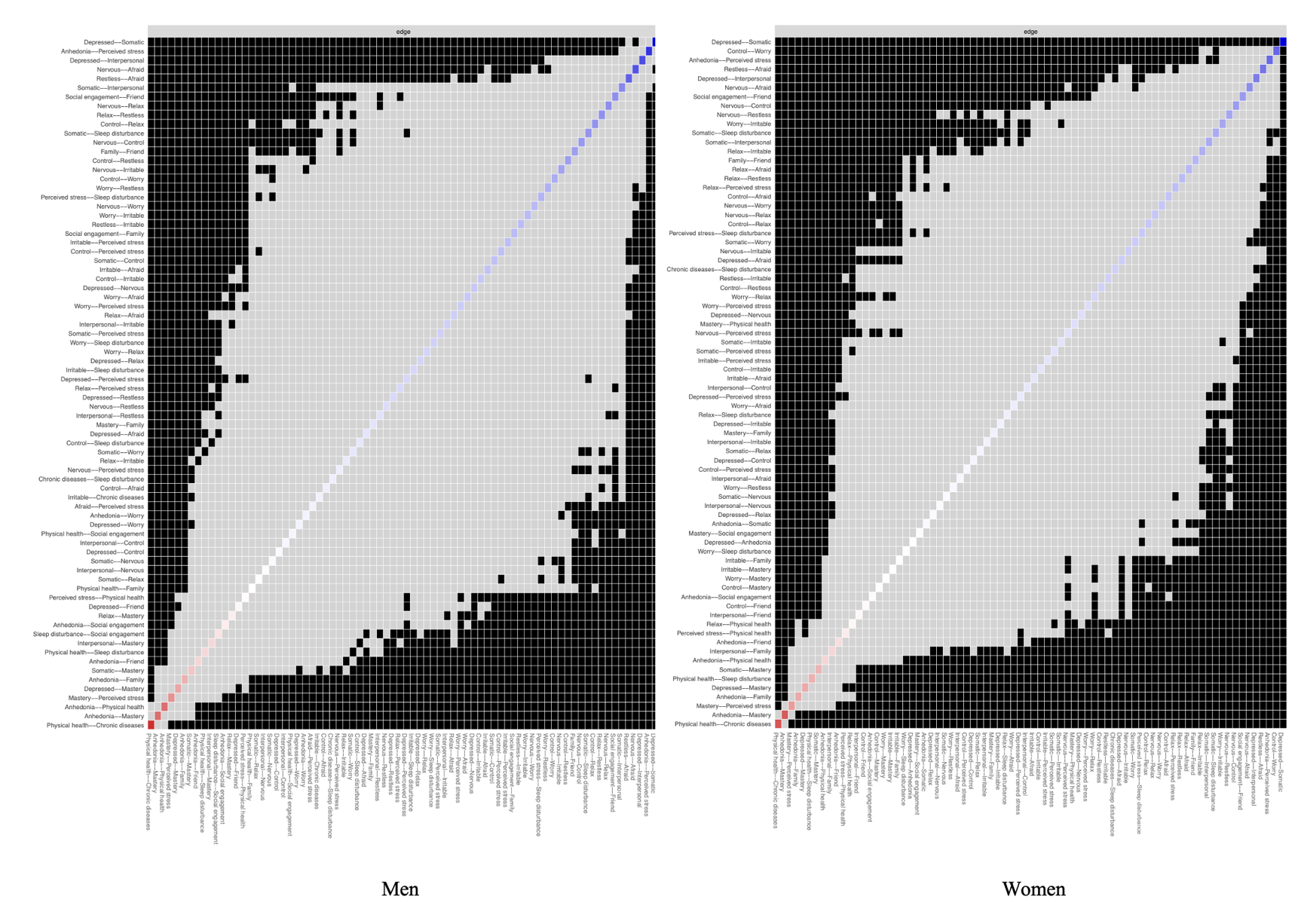


Figure S6

*Estimation of edge weight difference by bootstrapped difference test by gender*

Bootstrapped difference tests between edge weights. Black boxes indicate significant differences (α = 0.05) between edge weights, whereas gray boxes indicate non-significant differences between edge weights. Blue boxes in the edge-weight plot indicate positive correlations.


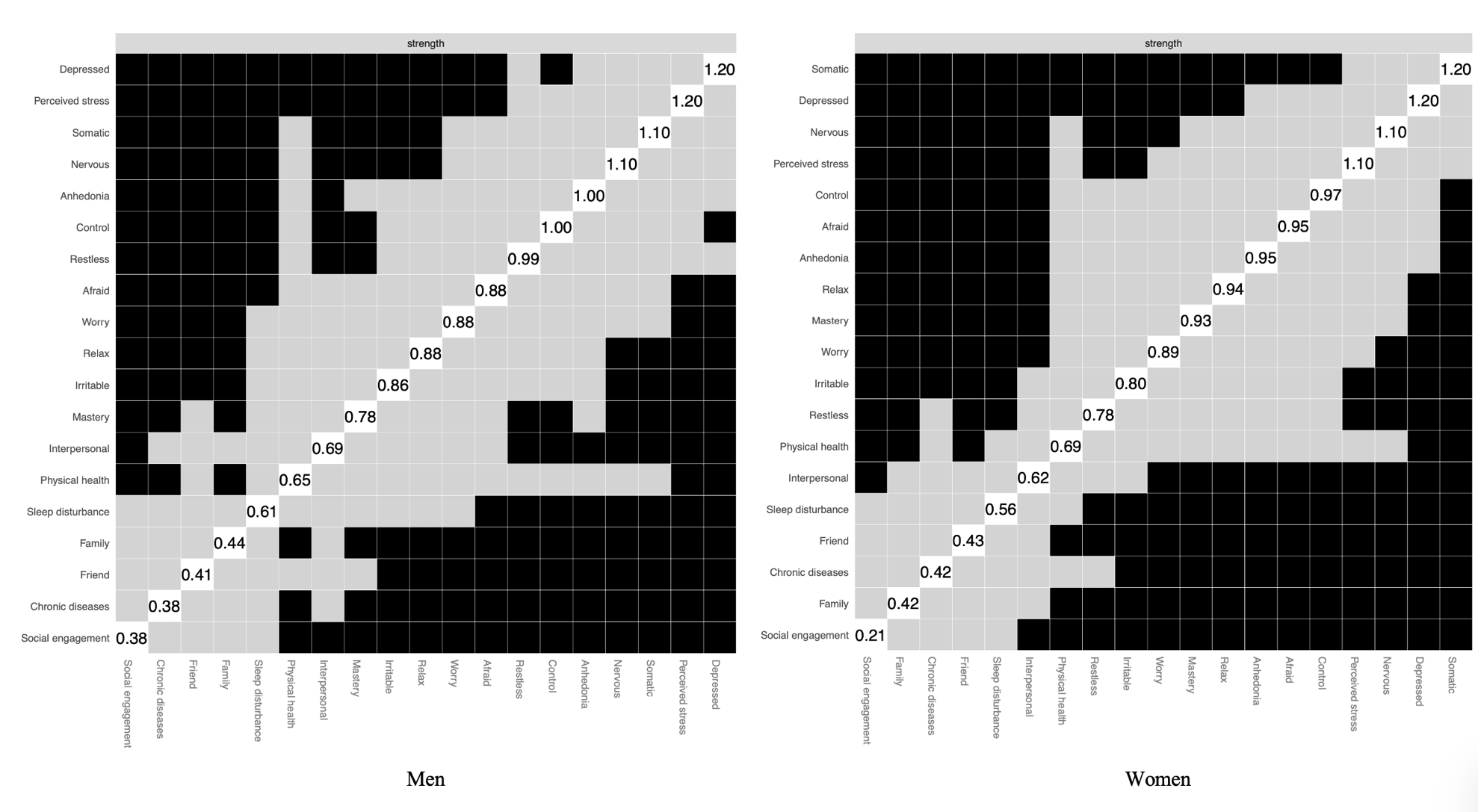


Figure S7

*Estimation of node strength difference by bootstrapped difference test by gender*

Bootstrapped difference tests between node strength of depression and anxiety symptoms, and physical, cognitive, and social factors. Black boxes indicate nodes that are significantly different from one-another (α = 0.05), whereas gray boxes indicate non-significant differences. Numbers in the white boxes indicate the values of node strength.

**
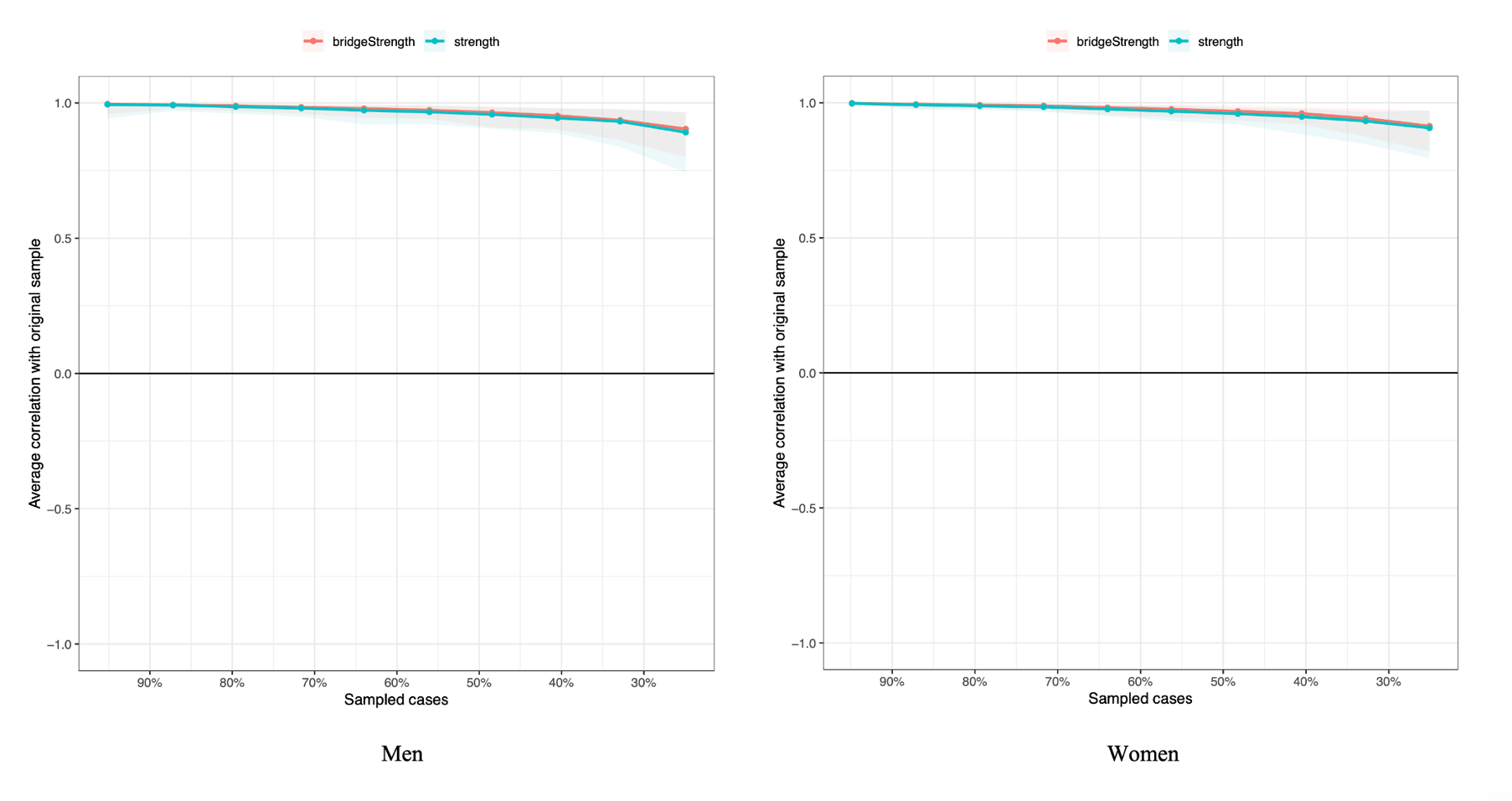
**

Figure S8

*Case-Dropping bootstrapped centrality indices by gender*

Red and blue lines indicate mean correlations between centrality values of original and sub samples when dropping different proportions of the data; Straighter line indicates more reliable centrality. Areas around the lines indicate 95% CIs. The plot of the estimated networks indicates stable and reliable centrality.
